# Qualitative insights into nurses’ emotional and functional experiences in the 2023 military humanitarian mission to Turkey

**DOI:** 10.1186/s13584-026-00747-2

**Published:** 2026-01-28

**Authors:** Sheli Shilman-Nomeisky, Angela Ruban, Irit Bluvstein, Ronen Segev

**Affiliations:** Department of Nursing Sciences, Gray Faculty of Medical & Health Sciences, The Stanley Steyer School of Health Professions, Tel Aviv University, Tel Aviv, Israel

**Keywords:** Emotional experience, Social functioning, Professional functioning, Nursing, Humanitarian aid delegations, Coping strategies, Disasters

## Abstract

**Background:**

Humanitarian aid delegations play a crucial role in assisting victims of disasters worldwide. Nursing and medical teams are involved in treating injuries and illnesses, promoting health, and providing emotional support to patients. Such teams often have long-term emotional, social, and functional impacts. Those impacts can include post-traumatic stress disorder, disrupted social relationships, and decreased professional efficacy. This paper examines the emotional, social, and professional experiences of nurses participating in the Israeli Defence Forces’ (IDF) 2023 humanitarian operation in Turkey and their coping mechanisms. It also examines their perceptions of how this experience may have influenced their response to the events of October 7th and the ongoing war.

**Methods:**

Twenty-seven nurses participated in a personal information form and the Life Events Checklist-5 (LEC-5) questionnaire, which evaluates previous trauma and life events. In-depth semi-structured interviews were conducted through Zoom. Data analyses were conducted using the seven-step method of the Colaizzi approach, and the research followed the COREQ guidelines to ensure comprehensive reporting and rigour.

**Results:**

The discussion has revealed four themes and ten subthemes, which were identified and grouped into the following categories: emotional well-being, social and professional functioning, and coping with disaster exposure. The participants’ narratives highlight their remarkable resilience. They were able to achieve emotional balance, address emotional difficulties, increase functional resilience, and cope with disruptions in their family life. Professional resilience, competence, and challenges at work were also discussed. Some of the coping mechanisms that were employed were the use of different types of support, self-control, and the use of psychological support. They recalled the effects of the October 7th events and the Iron Swords War on their lives. The participants provided information about how to deal with natural and human-induced disasters.

**Conclusions:**

The paper highlights the emotional, social, and professional implications of humanitarian aid for nurses and how they cope. To ensure that subsequent experiences with delegation are enhanced, it is crucial to provide continuous psychological support to delegates both during and after their missions. Equally important are operational improvements, such as creating support groups, hiring professional translators, and implementing straightforward operational protocols, which can significantly enhance nurses’ experiences.

## Introduction

Delegations of humanitarian aid are deployed in disaster zones, regardless of whether natural or artificial factors cause the disaster. The presence of these delegations is essential in saving the lives of the victims and alleviating their plight [1]. They are an integral part of disaster response, as they meet a large percentage of the needs of victims [2].

Since 1953 [3, 4], Israel has sent military and civilian humanitarian aid delegations to respond to crises like earthquakes, wars and terror attacks [5]. In February 2023, two significant earthquakes struck the Kahramanmaraş region, south-eastern Türkiye, killing approximately 57,000 people. In response, the Israel Defense Forces (IDF) dispatched a humanitarian delegation to provide assistance [6].

In various disaster areas, nurses play a crucial role as part of a response team [7]. Their duties cannot be ignored, and they include the process of triage, treatment of the injured, evacuation, and coordination with various organisations [8]. It is also the duty of nurses to protect victims, maintain their dignity, and offer support and advocacy [9]. In addition to patient outcomes, such teams face numerous consequences of their exposure to disasters, including emotional and functional outcomes [10, 11].

There can be both positive and negative emotional impacts on team members, both on missions and at home. Many studies have reported such adverse outcomes as burnout, stress, anger, lack of interest in work, personal subjective perception of low quality of life, depression symptoms, Post-Traumatic Stress Disorder (PTSD), anxiety, and addiction tendencies [10, 12, 13].

A study involving a Dutch humanitarian aid delegation to the 2010 Haiti earthquake, which consisted of 51 first responders, medical teams and communication personal. The study indicated that some of the participants experienced depression symptoms. However, three months after delegation, there was no significant difference in mental health between the time of delegation and the time of pre-assignment reports [14].

In a study examining anxiety, depression, and PTSD among international humanitarian workers, researchers assessed these conditions prior to the mission, upon their return, and two months thereafter. The findings indicated that anxiety levels were most pronounced before the mission, whereas the levels of depression and PTSD remained constant over time [15].

A qualitative study examining emergency response teams from the United States who participated in the 9/11 disaster response revealed that, even 15 years later, the event had not been forgotten and continued to exert a lasting impact, manifesting as guilt, depression, anxiety, PTSD, and sleep disorders [16]. However, other researchers have found positive emotional results, including personal and professional fulfilment, developing a system of support for victims [10], gratitude for being alive, and a desire to live better lives [16]. This paper examines the emotional experiences of nurses who participated in the IDF organized 2023 aid delegation to Turkey. Through in-depth interviews, we aim to gain a comprehensive understanding of their emotional experiences.

Besides the emotional consequences, humanitarian aid delegation staff members can face functional, social, and professional changes. For example, the emergency teams that participated in the 9/11 disaster attested to having reduced emotional abilities that influenced their personal lives, altered their dynamics at work, or led them to retire from their professional careers [16]. According to another study, exposure to traumatic workplace events was correlated with physical difficulties, worsening of social life quality and workplace mental issues [17]. Additional studies have suggested that employees who are affected by traumatic incidents and have PTSD are more impaired in their occupational and social functioning than employees who are not exposed to traumatic incidents [18]. The literature review revealed a lack of research on the emotional and functional effects of teams within IDF humanitarian aid groups.

The coping process is one of the obstacles in dealing with a disaster. The study of PTSD symptoms and coping mechanisms has recognised five coping strategies: active coping and engagement, social coping, coping by religious beliefs, repression, and coping by material substances [19].

Other coping mechanisms involve dependence on cultural factors, past experiences, and preoccupation with fundamental needs, i.e., obtaining food, water, shelter, and jobs [20]. Moreover, the study of Israeli nurses who have served in different Israeli wars revealed such strategies as seeking social support among workers and venting [21].

The other population analysed the Israeli people prior to the start of the Iron Sword War and a month after the start of the war, and found the number of reported symptoms of anxiety, depression and PTSD was more common after the events of October 7th. On October 7th, 2023, the Hamas organization launched an attack on Israel, resulting in the deaths of over 1,300 civilians and the abduction of 240 individuals who were subsequently held hostage [22]. This event precipitated the commencement of the Iron Swords War, signifying the initiation of Israel’s military response to these unprecedented assaults. To the best of our knowledge, very little research has been conducted to explore the coping strategies of humanitarian aid workers. Moreover, not many articles have addressed the coping mechanisms of the Israeli people in reaction to events on October 7th and the Iron Sword War.

In this regard, the current research examined the functional, social, and emotional transformations that nurses underwent during the IDF 2023 humanitarian operation in Turkey, as well as the coping mechanisms they employed. It also evaluated the effect of this experience on how they reacted to the events of October 7th and the ensuing war. Therefore, the following research questions were used:

In this regard, the current research examined the functional, social, and emotional transformations that nurses underwent during the IDF 2023 humanitarian operation in Turkey, as well as the coping mechanisms they employed. It also evaluated the effect of this experience on how they reacted to the events of October 7th and the ensuing war. Therefore, the following research questions were used:

1. What are the emotional and psychological effects that nurses experienced in IDF’s 2023 humanitarian aid delegation?
2. What are the effects on the social and professional functioning that nurses experienced in IDF’s 2023 humanitarian aid delegation?
3. How did the nurses who participated in IDF’s 2023 humanitarian aid delegation deal with or are they dealing with the emotional effects and the functional changes they experienced?
4. What are the perceptions of nurses regarding the influence of their involvement in the IDF’s 2023 humanitarian aid delegation on their ability to cope with the events of October 7th and the ongoing conflict?

## Methods

### Study design

The research methodology employed in this study was qualitative, involving semi-structured in-depth interviews. This methodological approach was chosen to provide a holistic picture of the participants’ experiences, thoughts, and perceptions regarding the topics being examined. The research was done with the permission of the Ethics Committee of the IDF medical corps and the Ethics Committee of Tel Aviv University (Approval No. 0009453-3). The participants signed a consent form prior to the interviews, thereby giving informed consent. Ethical standards regarding the confidentiality of participant information were strictly adhered to.

### Participants

The research included nurses who were part of the IDF’s humanitarian aid mission to Turkey following the 2023 earthquake. The study was voluntary, and participants were contacted by telephone using contact details initially provided by the former chief military nurse of the IDF, who was the head nurse of the delegation. Each participant was asked to consent to participate in the phone call and subsequently signed a consent form, ensuring their privacy was protected.

Throughout the analysis and manuscript preparation, participants were assigned numerical identifiers instead of their actual names. Identifiable data will be deleted upon completion of the research. The participants were asked to complete a questionnaire on personal details and another on life events.

Out of the 32 nurses who were invited to part in the delegation, 27 nurses agreed to join, including 13 men and 14 women with an average age of 43 years. Five interviewers did not participate, some declined to participate, and others had busy schedules. Nineteen of them held a master’s degree, and the rest held a bachelor’s degree. Also, 23 participants had taken higher nursing courses. The sample size is 17, with a mean professional experience of 17.18 years, and all 17 participants were working in hospital settings. Most importantly, 16 participants had never been part of any aid delegations before (Table 1).

**Table 1:** Participants’ characteristics (*n* = 27)

Variable	*N* (%)
Gender	
Male	13 (48.1%)
Female	14 (51.9%)
Average age (mean)	43 years
Marital status	
Single Married/In a relationship Divorced Other	4 (14.8%) 20 (74.1%) 2 (7.4%) 1 (3.7)
Children	
Yes No Number of children (mean)	20 (74.1%) 7 (25.9%) 2
Academic Degree	
B.A. M.A.	8 (29.6%) 19 (70.4%)
Professional education	
Without Advanced nursing courses Nurse practitioner	3 (11.1%) 23 (85.2%) 1 (3.7%)
Workplace	
Hospital Health maintenance organization IDF Other	17 (63%) 1 (3.7%) 5 (18.5%) 4 (14.8%)
Department	
Emergency department Pediatric emergency department Radiology department Intensive care unit Pediatric intensive care unit Operating rooms Dialysis department Recovery unit Administrative role IDF departments Other	3 (11.1%) 1 (3.7%) 1 (3.7%) 5 (18.5%) 1 (3.7%) 2 (7.4%) 1 (3.7%) 1 (3.7%) 3 (11.1%) 5 (18.5%) 4 (14.8%)
Employment status	
66% 88% 100%	1 (3.7%) 4 (14.8%) 22 (81.5%)
Nursing seniority (mean)	17.18 years
Religion	
Jewish Druze Other	25 (92.6%) 1 (3.7%) 1 (3.7%)
Level of religiosity	
Secular Traditional Religious Other	16 (59.3%) 5 (18.5%) 2 (7.4%) 4 (14.8%)
Number of delegations	
1 delegation 2 delegations 4 + delegations	16 (59.3%) 7 (25.9%) 4 (14.8%)
Evacuation after October 7th events	
Yes No	1 (3.7%) 26 (96.3%)
Military mandatory/reserve service Reserve Mandatory No service	18 (16.77%) 5 (18.5%) 4 (14.8%)
Loss experience after the October 7th events	
Yes No	18 (66.7%) 9 (33.3%)
LEC-5- events that happened to me (mode)	4 (23.5%)
LEC-5- events that happened to me, witnessed id or learned about it (mode)	15 (29.4%)

The interviewer was a female with a B.A. degree in Nursing and is a M.S.N. candidate in Nursing Sciences. She works at Maccabi Health Maintenance Organization as a nurse in multiple departments. There was no previous relationship between her and the participants. The interviewer’s professional experience could facilitate mutual understanding and closeness to the participants. To minimize bias, the interviewer made reflexive comments after each interview, and each interview was analyzed separately.

## Materials

**Fig. 1:**
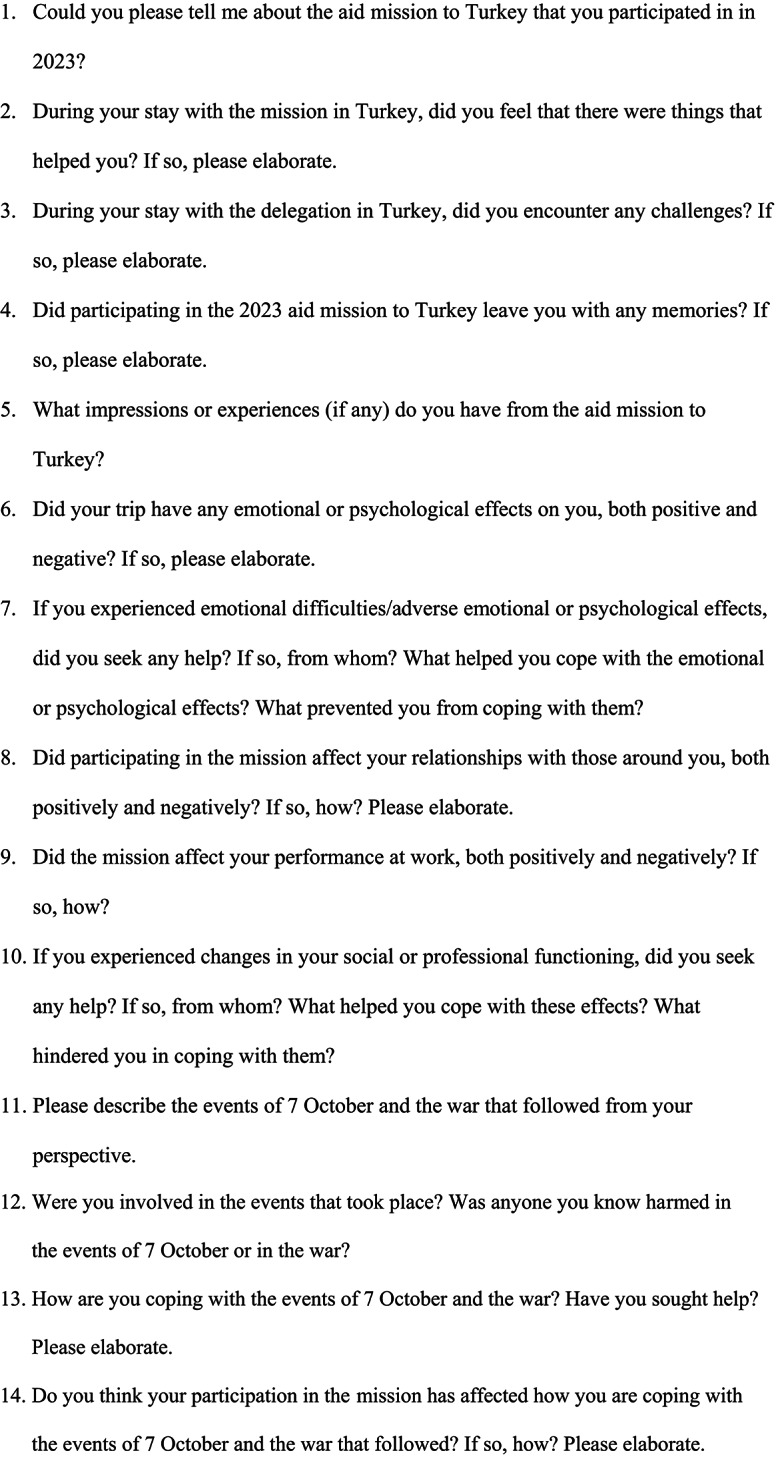
Interview Guide

The guide was created by the researchers, based on the research questions. The participants were asked to answer open ended questions about their experiences about the delegation process. They gave comprehensive reports of any difficulties, emotional effects, and functional adaptation to the delegation experience during and after and how they coped with them. They were also asked about what occurred on October 7th, and the conflict that followed, whether they were involved in the events, how they coped with the events, and whether they thought their coping was made easier by the fact that they were a part of the delegation (Fig. 1).

Life Events Questionnaire:

To determine prior traumas, the participants were asked to fill out the Life Events Checklist (DSM-5) of LECS. This is a tool that evaluates exposure to 17 events that can cause PTSD [23]. Two certified translators were used to translate the questionnaire with the translation and back-translation method. Three judges, which included a psychologist and two certified nurses, carried out the final evaluation, also being the authors of this study. Based on the LEC-5 results, a majority of the interviewees were already aware of four events in the list, and a majority had experienced, observed, or heard 15 events (Table 1).

### Data collection

The interviews were conducted via Zoom to overcome the time and space limitations of face-to-face interviews. These were carried out between December 2024 and March 2025 and lasted an average of 45 min. The participants decided on the setting they preferred, either at home or at the office, and dedicated time for the interview. All were interviewed privately, without others present or interruptions, by the researcher and the participants.

The sessions were tape-recorded to facilitate easier transcription and analysis of the content. Both audio and video recordings were utilized in this study. Participants were duly informed about the recording process prior to the interviews and provided their consent. To ensure privacy, the recordings were stored on a secure, password-protected server, accessible exclusively to the research team. During transcription, any identifying information was removed, and the transcripts were anonymized before analysis. Field notes were taken during the interviews; no repeat interviews were conducted. The first author took the interviews.

To prevent overburdening all participants, particularly given the challenges of scheduling and their limited availability, a selective member-checking process was implemented. This process involved a subset of participants, specifically 10 out of 27, who were chosen to represent diverse backgrounds, including professional education, gender, marital status, and academic degree. These participants received their interview transcripts for verification and provided feedback, which was subsequently incorporated to enhance the credibility and accuracy of the analysis.

The potential for participant distress in trauma-oriented research is extensively documented in the literature. To mitigate this, all interviews were conducted in accordance with established distress protocols for qualitative research. These measures included pausing interviews upon observing signs of distress, considering terminating the interview if necessary, and offering access to professional mental health support [24].

### Data analysis

The analysis of the data was performed according to the seven-step approach presented by Colaizzi and the COREQ guidelines [25, 26]. Initially, the transcripts of the interviews were read several times to gain a comprehensive understanding. At the next step, the important sentences were singled out, and meanings were then elicited from these sentences to create a list of codes. There were 772 distinct codes in the first analysis, which the first author analysed.

The fourth step involved categorizing codes into themes based on their similarities, with the development of different themes dependent on the research questions. The fifth step entailed recording the results in terms of the themes. The themes and subthemes were reviewed by the first and last authors, who analysed the data separately and then discussed the findings with the third author to reach a consensus. The researchers approved the accuracy of the collected data and the final division.

The sixth stage involved refining the descriptions to emphasize the importance. The quotes were selected and reviewed in accordance with the chosen themes and subthemes. It was discussed with a few participants in the final stage to ensure consistency with their insights; they received them and commented on their thoughts [25].

## Results

**Fig. 2:**
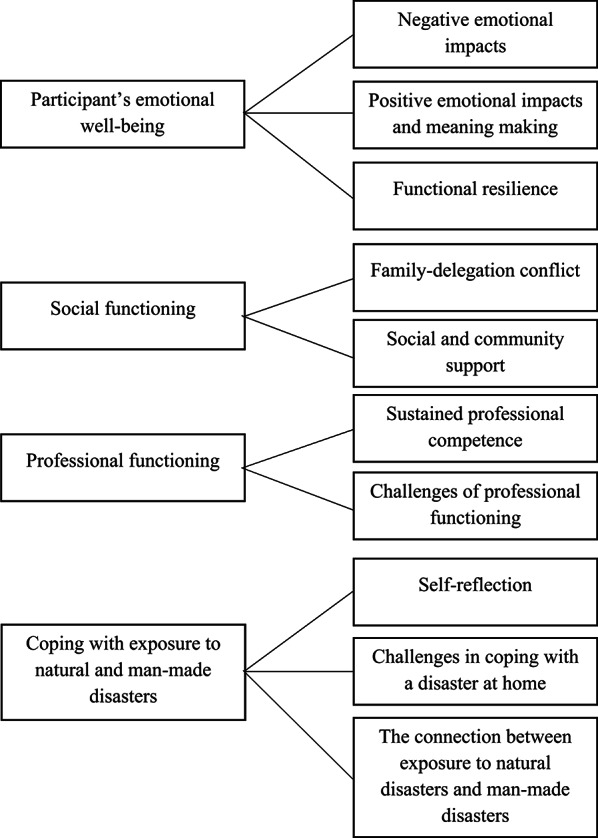
The study themes and subthemes

The research aimed to clarify the subjective views of the participants regarding the emotional, social, and professional consequences they faced, as well as the coping strategies they used in response to earthquakes and war. The themes were based on the analysis of four main themes and ten subthemes (Fig. 2).

### Theme 1: participants’ emotional well-being

Participants are likely to experience a range of emotional effects during and after the delegation. These effects may be detrimental, potentially disrupting coping mechanisms and exacerbating stress levels. Conversely, they may also be beneficial, contributing to well-being, fostering positivity, and enhancing interpersonal relationships.

### Negative emotional impacts

The disaster had significant psychological impacts on the nurses, as they were required to confront its repercussions, manage uncertainty, and contend with unsuccessful outcomes.

Participant 5 expressed the feeling of devastation after knowing the destiny of the victims:

*“You think about the kids we took care of there and their parents dying*,* and you leave your kids at home to fly to take care of other children that don’t have parents*,* you don’t know how they will get along*,* it really hurts your heart”.*

Participant 13 described the feeling of helplessness while trying to help one of the victims and not being able to do so:

*“It’s a terrible feeling of helplessness. You are the one who broke someone’s last piece of hope. I didn’t cause the earthquake*,* but I was part of the people who told him that his world was destroyed. It’s a very hard feeling”.*

While preparing for the delegation and upon arriving there, he also mentioned the feeling of uncertainty:

*“I don’t know where to throw my stuff*,* when I’ll be coming back*,* or where I’ll be sleeping. There were a lot of moments during the flight of uncertainty*,* when we would leave*,* and how. There were difficult moments; it was very cold and rainy. Where will we go*,* where will we work? There was a lot of uncertainty”.*

***Positive emotional impacts and meaning making***.

In addition, many interviewees experienced positive emotional impacts both before and during the delegation process. The effects are still felt today as a reminder of the role that their wish to help others, feelings of gratitude and knowledge acquired can play in their well-being. Participant 10 described the excitement he felt even prior to the delegation and his excitement to help the injured:

*“I immediately said yes*,* with great excitement*,* with such an emissary feeling. You don’t think about anything*,* not about the dangers*,* the concerns*,* the fears*,* not about what will be with you. You just want to be a part of some human act and to help those in need”.*

Participant 26 described feeling valued by his family, his colleagues, and the locals and feeling meaningful by helping others:

*“The experience is very powerful*,* meaningful*,* and impacts your thinking pattern. You are in a place where you feel very valued*,* by your colleagues at home and your family*,* which is very empowering personally”.*

Participant 22 highlighted the distinct nature of his experience and the insights gained from it:

*“A unique experience. Every event teaches you something; you don’t get to do that a lot in life. The things we did there were very positive and contributed to the community”.*

### Functional resilience

Participants reported enhanced functional resilience from their involvement in the delegation. They expressed a sense of appreciation and affection both during and subsequent to the delegation, directed towards themselves and their loved ones.

Participant 1 expressed a sense of pride in taking part in the delegation and reported receiving positive feedback from his surroundings, which he felt enhanced his resilience:

*“It was nice that my father had something to be proud of; you always want to make your parents proud and happy. My manager also felt pleased. I got messages from my colleagues all the time; they were proud of me too”.*

Conversely, participant 11 articulated a perceived necessity to express greater appreciation for his family, which subsequently fortified their relationship:

*“You have got to appreciate what you’ve got. Only when you see such things do you truly appreciate them. We think we appreciate*,* but in reality*,* we don’t. When you come back from there*,* you tell yourself: relax*,* handle your life a little differently. You can’t wait to get home and truly appreciate your house and your family”.*

Participant 13 reinforced those feelings, and even felt the necessity to live every day like it is the last one:

*“I think that the experience of dealing with such a situation left an imprint on my brain to remember that those are your friends and family. It could happen every minute; no one will prepare you for that. I may be exaggerating a little*,* but you need to live every day like it’s your last day”.*

### Theme 2: social functioning

Being a part of a humanitarian aid delegation can also impact negatively on social functioning and relationships with family, friends and surroundings. Those impacts can affect one’s social functioning negatively. To cope with the impacts, nurses can rely on support from their surroundings.

### Family-delegation conflict

Some participants experienced negative social outcomes, especially concerning their relationship with their families. Some of the participants described difficulties they had with their families regarding participation in the delegation.

Participant 14 described having an argument with a family member about joining the delegation:

*“Some didn’t like that I went on the delegation. I even had an unpleasant argument with my dad before that; he didn’t think it was right for me to go”.*

Participant 5 talked about it too:

*“I really wanted to go to the delegation and I was happy that I got picked to go. It was important to me personally. I even fought with my husband*,* he didn’t want me to go because Turkey is a hostile country*,* and I said I was willing to do everything in order to go”.*

Participant 24 described the sacrifice of going on the delegation, and how it affects family life:

*“Every departure to a delegation is a challenge. It always comes when you have something. For us*,* it was at the same time as a family vacation we had for our grandson’s Bar-mitzva (age 13 Jewish boy’s ceremony)*,* and I missed it. But it always comes out either on Saturday night*,* or on holiday eve*,* or on other events”.*

### Social and community support

In various contexts, receiving support from colleagues has helped manage challenges. Participant 3 articulated a coping strategy that relies on social support within the team: *“The team*,* the human part*,* you can deal with all the technical challenges if you have good people beside you. You can handle all of the mental struggles better if you have people whom you can share with what you experience”.*

Similarly, participant 12 emphasized the importance of daily debriefing sessions for mutual sharing and reflection: *“At the end of every day*,* we held a briefing. Everyone spoke*,* whether about a patient or about themselves*,* because everyone needed the space to share what they thought and felt”.*

In addition to peer support, professional assistance was consistently available throughout the delegation. The team was accompanied by a mental health officer who facilitated emotional check-ins and established a safe environment for sharing:

A mental health officer accompanied us. He inquired about our well-being, and everyone shared as much information as we desired.

### Theme 3: professional functioning

In addition to the emotional and social effects, participation in a delegation can significantly impact the team’s occupational functioning during the delegation. Nurses may experience professional success in this context; however, they may also encounter various professional challenges.

### Sustained professional competence

In terms of occupational functioning, numerous nurses reported experiencing positive impacts and achieving success in their professional roles.

Participant 21 recounted his effective management of work responsibilities within the delegation.

*“We did a lot*,* we reactivated a local hospital that wasn’t functioning*,* got it moving from zero to one*,* and helped it start operating again. The local team slowly agreed to return to work. We started the return of the routine there”.*

Participant 11 described working in the best way he could and according to high standards.:

*“We worked according to the highest standards. We didn’t do anything we hadn’t done before or endanger anyone. We did everything at the highest level*,* no matter what terms you worked in*,* because we couldn’t allow ourselves to hurt anyone”.*

Participant 10 articulated a sense of purpose in being valuable to the patients and in applying his expertise and skills to achieve optimal outcomes.

*“Once you know you can be helpful with your knowledge and skills*,* once you are surrounded by a society that accepts and allows it*,* you feel empowered*,* strong*,* and helpful*,* you feel connected to the aim of the delegation. That is what we experienced”.*

### Challenges of professional functioning

Besides their career success, the nurses mentioned that they have faced several challenges, including adjusting to different work settings, the need to cooperate with diverse individuals, and overcoming obstacles.

Participant 11 specifically highlighted the challenges of managing foreign employees.

*“Every day there was a new team. You work for a day or two with a crew*,* then they go*,* and a new team comes. You already got used to the people*,* you formed communication with them*,* and then it goes again. For some of the people it was very difficult*,* it was like being on a different work every day”.*

Participant 18 described the difficulty of working in a different working setting:

*“In the professional field*,* we weren’t the ones to develop the capabilities and to define the work methods. We came to something that existed (worked in a local existing hospital in the disaster zone) and integrated with the local teams. It was a challenge to know the equipment*,* the working protocols*,* and the work environment”.*

Another important challenge that participant 10 had to cope with was the language, a huge part of the nurse’s work:

*“The nurses really like to talk*,* to explain*,* to guide*,* to comfort*,* and to help. Suddenly a very important tool that you use every day as a nurse is taken away from you. It was very hard for me as I don’t understand Turkish”.*

### Theme 4: coping with exposure to natural and man-made disasters

In addressing various types of disasters, coping strategies can differ significantly. Some individuals may rely on personal attributes, while others may draw upon their prior experiences to aid their coping mechanisms. However, disasters occurring within one’s own country often present greater challenges in terms of coping with them.

### Self-reflection

Several participants addressed both natural and anthropogenic disasters by leveraging their personality traits and managing their emotions effectively.

Participant 9 specifically mentioned relying on these strategies during delegation:

*“I think that in my personality structure*,* I am probably very resilient. I went into an automaton mode all those days”.*

Participant 13 mentioned using humour and cynicism while dealing with the earthquake:

*“In an attempt to adopt a cynical perspective infused with self-deprecating humour*,* I uploaded a photograph capturing the initial evening spent with the sleeping bag and tent*,* accompanied by a cup of tea placed on the ground and a pair of gloves. I composed a cynically humorous caption*,* which*,* while not universally amusing*,* was intended to be entertaining*,* akin to a five-star hotel. My intention was to preserve this moment as a source of personal amusement”.*

Participant 11 articulated the notion that personal transformation is a prerequisite for broader societal change:

*“I realised that to effect change in the world*,* one must first undergo self-transformation. It is imperative for individuals to prioritize the cultivation of their character*,* to strive towards moral integrity and humanity*,* and subsequently*,* the surrounding world will begin to transform.”*

### Challenges in coping with a disaster at home

Many of the participants vividly described the emotional turmoil they faced in dealing with the events of October 7th and the Iron Swords War, a disaster that struck at the heart of their home.

Participant 25’s story is a testament to the resilience of the human spirit in the face of trauma.

*“Every day*,* I find it hard to disconnect from October 7th and what happened after it*,* from the soldiers who were killed and their families. This is an ongoing challenge. I believe the world is divided into two groups: those who are disconnected and those who are connected. Maybe because I am on reserve duty*,* I feel that way”.*

Participant 7 expressed difficulty in comprehending and accepting the situation, stating,

*“Even a year later*,* I occasionally struggle to believe that this is what we experienced. I still find myself questioning*,* ‘Did it really happen? Is it still happening?‘“*.

Similarly, Participant 25 articulated a strong desire to assist, coupled with a sense of helplessness due to inaction:

*“I was waiting for someone to request my help*,* as that was my intention. I felt helpless merely sitting there*,* not engaging in any activity*,* not assisting or contributing.”*

#### The connection between exposure to natural disasters and man-made disasters

Perspectives on the relationship between participation in the delegation and involvement in the Iron Swords War were diverse. Some participants perceived no connection between the two events, whereas others speculated that a link might exist.

Participant 1 articulated a distinction between these events:

*“I cannot compare them. In the delegation*,* although my empathy extended to a population not directly related to me*,* it was directed towards individuals who were fundamentally similar to us. In contrast*,* the events here affected individuals who served alongside me*,* individuals who are soldiers in the same organisation as I am. This situation possesses different dimensions; it is much more immediate and challenging to comprehend.”*

Participant 5 articulated the notion that one can derive learning from any experience. He perceived that the delegation experience enhanced his ability to contextualise situations, a skill further augmented by his wartime experiences:

*“I believe that every place you visit provides you with tools to manage your experiences. During the delegation*,* I endured a week without showering*,* eating*,* or changing clothes*,* which puts the experience of returning home every few days while stationed at a military base during the war into perspective. This process allows one to contextualise everything in the data”.*

Participant 15 shared their personal insights on the influence of specific personality traits on an individual’s decision to participate in delegations and their ability to navigate challenging situations:

*“I believe that*,* given my personality structure*,* if I were not resilient*,* I would not have considered participating in delegations from the outset*,* and my coping mechanisms might have differed.”*

## Discussion

In this paper, the authors explore the perceptions of the emotional, social, and professional experiences of nurses involved in the IDF’s humanitarian assistance delegation to the 2023 Turkey earthquake. The study investigated participants’ reflections on their coping strategies and their perceptions regarding whether and how participation in the delegation may have influenced their responses to the events of October 7th and the subsequent war.

Some participants in the delegation work complained of negative emotional impacts, including sadness upon witnessing the disaster’s impact on people, feelings of helplessness when unable to assist, and uncertainty about the future. Other studies conducted previously have also reported a sense of helplessness [27], as well as sadness and frustration, among medical staff in disaster areas [28]. Another issue encountered by these teams is the need to make decisions in the face of uncertainty [29].

On the contrary, some nurses also experienced positive emotions, feeling appreciated by the local population, having a strong sense of purpose, being strongly motivated to help others, and having meaningful learning opportunities. Many nurses received positive reinforcement, as the communities they lived in were proud of and supported them.

The interviewees discussed improved relationships and a newfound value in life. It has been mentioned in previous studies that aid teams usually feel valuable and welcome [10], and victims can also show their gratitude to their assistants [21]. In this case, nurses are also more likely to develop a sense of urgency and desire to help [30, 31].

Health policymakers have highlighted various approaches to manage these effects and enhance participants’ positive emotional outcomes. Participants indicated that peer support was advantageous, suggesting that structured support groups may be beneficial and warrant further investigation.

Using those approaches, as described above and below, both nationally and internationally, can help participants deal with adverse outcomes and enhance positive ones.

Socially, some nurses also reported adverse effects, including social strain, conflicts with loved ones, and the absence of essential events due to work commitments. Similar family tension has been observed in relation to aid missions [30] and the adverse social impact of exposure to trauma [17, 32].

As coping mechanisms to all these negative impacts, participants employed the support of teams, daily debriefs, and support services of mental health professionals. The effectiveness of social support [9, 19, 21, 33], professional mental health care [34], and debriefing in developing resilience and enhancing coping processes is effective [29].

As noted by the participants, health policy stakeholders recognise and utilise such strategies to enhance the social impact on teams. Providing the support systems before, during and after the delegation can also help the healing process. The support systems offered to the strained families can also help enhance their performance [35]. Based on the participants’ experiences, improving communication between them and addressing challenges can benefit both participants and their families.

Nurses explained the beneficial and harmful effects on their careers. Others were quite useful and constructive, utilising their expertise, working to high standards, and benefiting the local people. Research has revealed that a disaster working environment can facilitate job satisfaction [36]. On the other hand, some were interrupted in their routine and had communication difficulties.

The problems discussed in the literature include uncertainty in conflict zones [37]. The health policy includes a training program that entails training in different disaster scenarios to enhance preparation, backed by previous research [38, 39]. Team preparedness can be improved by adding various operational protocols to the training programs. Additionally, having a higher number of professional translators from different countries, combined with the application of cultural sensitivity in training, may also positively impact the results. The development of global cooperation and practice may help the performance of the team [40]. The humanitarian deployment must also be viewed as a component of the national health and workforce development policies of every country, rather than as a means to promote global cooperation and best practice.

The team also employed self-regulation strategies, which included relying on personal characteristics and inner strength to help them deal with the effects of delegation, in line with the coping theory formulated by Folkman and Lazarus [41].

Participants expressed feelings of distress and sadness. Their sense of regret stemmed from the tragic events of October 7th and the ongoing conflict, primarily due to their perceived inability to provide assistance or alleviate suffering during that critical period.

Studies have revealed that war-related disasters in the community of an individual may lead to depression, fear and stress [22, 34]. Psychological support for nurses, attending to their needs, and peer support groups may prove effective in various disaster situations [34, 42].

Regarding coping with war, participants mentioned that the earthquake was emotionally detached compared to the conflict taking place in the country. Others pointed out that their career experience gave them a broader outlook and better stress management. Some of the interviewees attributed their personality factors as the reasons why they had the motivation to get involved and developed coping skills.

To date, there is no research on the populations affected by earthquakes and war. The study of what participants learned about coping with various disasters can inform the design of specific support systems and training interventions [34, 38, 39, 42]. Health policies nationally and internationally can involve various strategies that would improve the experiences of teams during different disasters and lead to healthy coping strategies.

### Strengths and limitations

Several significant limitations of this study must be acknowledged when interpreting the findings. Firstly, the sample comprised nurses from a single humanitarian delegation organized by the IDF in response to the 2023 earthquake in Turkey. Consequently, the findings reflect context-specific experiences influenced by organizational structure, military affiliation, cultural background, and the specific nature of the disaster response. Therefore, the transferability of these findings to other humanitarian settings, professions, or countries is limited.

Secondly, the data are derived from retrospective self-reported perceptions, which may be subject to recall bias, personal interpretation, and subsequent events, including the October 7th attacks and the ongoing conflict. The study does not permit causal conclusions regarding the impact of delegation participation on coping, resilience, or professional functioning.

Thirdly, although efforts were made to enhance rigor through reflexivity, member checking with a subset of participants, and adherence to COREQ guidelines, researcher interpretation remains an inherent aspect of qualitative analysis.

Despite these limitations, the study offers rich, in-depth accounts of nurses’ lived experiences and identifies themes that may inform future research, hypothesis generation, and the development of context-sensitive support frameworks for humanitarian healthcare workers.

### Recommendations for health policy leaders

In addition to the aforementioned strategies, health policy stakeholders are encouraged to consider the following suggestions to enhance the effectiveness and well-being of future delegations. There is an imperative need for further research into the topics discussed. The recommendations derived from this study can be utilized to improve the preparedness, performance, and psychological safety of humanitarian teams. Concurrently, broader recommendations from the literature aim to fortify the professional identity and motivation of the participating nurses. These recommendations can be integrated into the standards of disaster nursing in Israel and globally, thereby fostering international partnerships and collaborative practices with various countries, which will facilitate the achievement of these objectives.

## Conclusions

This qualitative study provides a comprehensive analysis of nurses’ subjective experiences following their involvement in the 2023 IDF humanitarian aid delegation to Turkey. Participants reported a complex interplay of emotional distress, personal meaning-making, social challenges, and professional reflections. These narratives illustrate how nurses interpret and make sense of humanitarian work, rather than demonstrating objective or universal outcomes.

While some participants perceived their delegation experience as informative for coping with subsequent national crises, such perceptions should not be construed as evidence of preparedness or resilience enhancement. Instead, the findings highlight the necessity for structured psychological support, clear operational frameworks, and ongoing organizational attention to the well-being of humanitarian healthcare workers.

Further research across diverse settings, with longitudinal designs and comparative groups, is required to better understand the longer-term emotional, social, and professional implications of humanitarian deployments.

## Data Availability

The datasets generated and/or analysed during the current study are not publicly available due to the privacy of the participants but are available from the corresponding author on reasonable request.

## Abbreviations

IDF: Israeli Defense Forces
LEC-5: Life events checklist 5
PTSD: Post-Traumatic Stress Disorder
